# The impact of COVID-19 on eating disorders: A longitudinal study with assessments before and after the lockdown

**DOI:** 10.1192/j.eurpsy.2021.283

**Published:** 2021-08-13

**Authors:** E. Rossi, E. Cassioli, G. Castellini, G. Sanfilippo, F. Felciai, A.M. Monteleone, V. Ricca

**Affiliations:** 1 Psychiatry Unit, Department Of Health Sciences, University of Florence, Florence, Italy; 2 Department Of Psychiatry, University of Campania “Luigi Vanvitelli”, Naples, Italy

**Keywords:** eating disorders, post-traumatic stress disorder, COVID-19, quarantine

## Abstract

**Introduction:**

The COVID-19 epidemic that spread in Italy in the early 2020, together with the general lockdown, are high-risk events for vulnerable populations who need high levels of assistance, such as patients with eating disorders (EDs).

**Objectives:**

To evaluate the impact of the COVID-19 epidemic and lockdown on subjects suffering from EDs, considering previous vulnerabilities.

**Methods:**

74 patients with anorexia nervosa (AN) or bulimia nervosa (BN) already on treatment and 97 healthy controls were evaluated between November 2019/January 2020 (T1), and again in April 2020, 6 weeks after the start of lockdown (T2). Patients were also evaluated at baseline (T0). At each assessment, general and ED psychopathology (SCL-90-R and EDE-Q) were assessed. Childhood abuse experiences (CTQ) and adult attachment (ECR-R) were investigated at T1, and post-traumatic stress symptoms (IES-R) at T2.

**Results:**

Patients reported a significant increase in compensatory exercise; in addition, patients with BN and those who achieved remission at T1 showed a significant exacerbation of binge-eating. The longitudinal trend (T1-T2) of psychopathology was not different between patients and controls, however the expected benefit from treatment on ED psychopathology was significant only for AN, while no changes were noted in BN. Patients with BN reported more severe post-traumatic stress symptoms than AN and controls, and these symptoms correlated positively with prior traumatic experiences and an insecure attachment style.

**Figure:**
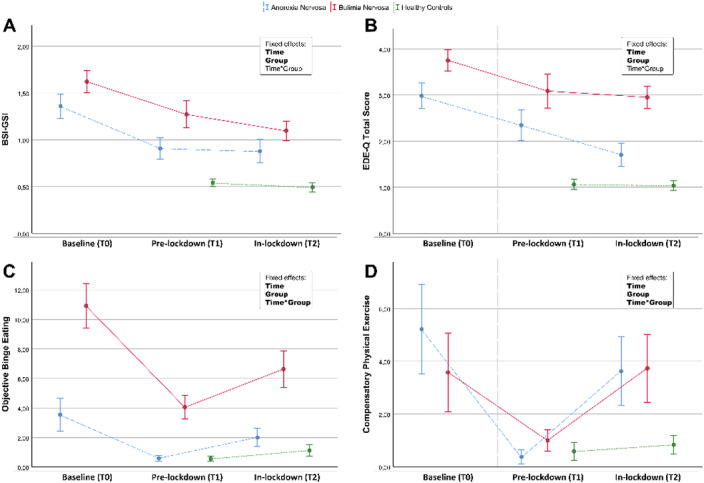

**Conclusions:**

The COVID-19 epidemic and lockdown had a significant impact on subjects with eating disorders, both by interfering with the treatment process and in terms of post-traumatic stress symptoms.

**Disclosure:**

No significant relationships.

